# Caries Risk Assessment in School Children Using Reduced Cariogram Model

**DOI:** 10.12669/pjms.334.13106

**Published:** 2017

**Authors:** Muhammad Taqi, Ishak Abdul Razak, Norintan Ab-Murat

**Affiliations:** 1 Muhammad Taqi, MSc. Department of Community Dentistry, Dow Dental College, Dow University of Health Sciences, Karachi, Pakistan, Department of Community Oral Health & Clinical Prevention, Faculty of Dentistry, University of Malaya, Kuala Lumpur, Malaysia; 2 Ishak Abdul Razak, PhD. Department of Community Oral Health & Clinical Prevention, Faculty of Dentistry, University of Malaya, Kuala Lumpur, Malaysia; 3 Norintan Ab-Murat, PhD. Department of Community Oral Health & Clinical Prevention, Faculty of Dentistry, University of Malaya, Kuala Lumpur, Malaysia

**Keywords:** Caries risk assessment, Cariogram, Children, Dental caries

## Abstract

**Objective:**

To estimate the percentage of children with low, moderate and high caries risk; and to determine the predictors of caries risk amongst 11-12 year old Pakistani school children.

**Methods:**

Subjects’ caries risk was assessed using the Cariogram programme. The survey was done among school children in Bhakkar district of Punjab, Pakistan. Caries and plaque level were assessed using the DMFT and Sillnes and Loe indices respectively, while diet content and frequency were assessed using a three-day diet diary.

**Results:**

A total of 226 children participated in this study, giving a response rate of 75%. Most subjects (39.8%) were in the low risk category with DMFT score of 0. The overall mean DMFT score was 1.49 (±0.63). Linear and multiple regressions were performed to evaluate the predictability of caries risk. Previous experience of dental caries was found to be the most relevant predictor of caries risk (40%).

**Conclusion:**

The levels of caries risk amongst Pakistani 11-12 year old school children were generally low. Past caries experience was the most significant factor in predicting future caries lesion in this sample population.

## INTRODUCTION

Caries risk assessment (CRA) is a critical part of a patient-centred caries management.1 The procedure assists clinicians in selecting the appropriate management based on individual’s caries risk level and to decide on the appropriate recall interval. A computerized programme for caries risk assessment known as Cariogram, has been shown to be effective in evaluating caries risk.1, 2 The programme was developed to demonstrate the link between caries and its associated factors, to clarify the chance to avoid caries, to produce a graphical presentation of caries risk, and to prescribe preventive treatments. It somewhat demonstrates an over-all risk situations.3 The application of full Cariogram requires bacterial and saliva testing which may not be feasible in epidemiological surveys as they are expensive and immediate results are not possible. The delay may affect patient’s motivation.4, 5 The use of reduced cariogram, by eliminating laboratory tests, has been shown to provide similar predictive caries risk as the conventional Cariogram. Moreover, evidence exists to show that reduced cariogram can be used to identify caries risk in preschool and school going children.4-6

The government of Pakistan spends 2.8% of its annual budget on health. Almost a quarter of the country health care cost is borne by the public health sector while the remaining 77% is financed through out-of-pocket payments in the private health sector. A pathfinder survey reported a caries prevalence of 50% among 12-15 year old Pakistani children.7 Small scale recent cross sectional studies conducted in selected cities in Pakistan also reported a caries prevalence of between 40 – 55% in 12-year-old children.8, 9 As the prevalence of caries among Pakistani 12 year old is quiet high and there is lack of resources to manage the condition, classification of children to different caries risk level would enable appropriate and cost effective caries intervention strategies to be employed on this population.

The objectives of this study were to estimate the percentage of children with low, moderate and high caries risk, and to evaluate the risk factors that largely predict the caries risk profile of 11-12 year old Pakistani school children.

## METHODS

This was a cross sectional study involving 11-12 year old Pakistani children. Sample size for this study was determined on the basis of previous prevalence study.10 Using standard error of 5%, a confidence interval level of 90%, and 5% attrition rate, the minimum sample size required for this study was 226. This study was approved by the Ethics Committee of the Faculty of Dentistry, University of Malaya and the permission to conduct this study in school premises was obtained from the administration of each school.

This survey was undertaken in seven schools (2 rural and 5 urban) located in the Bhakkar District of Punjab, Pakistan. The inclusion criteria were school children aged 11 to 12 years old with parental consent. The exclusion criteria were subjects who had no parental consent, had mental or physical disabilities or who were undergoing orthodontic treatment. Participants with signed consent were selected for risk assessment. After risk assessment, stratification was performed according to the risk category and then all participants were invited to participate.

Caries status was assessed using the DMFT index and plaque level was assessed using the Silness and Loe index. The calibration exercise for the DMFT and plaque index was performed on 20 subjects at an interval of two days. Intra examiner reliability was 0.88 indicating substantial agreement. Intra oral examinations were performed in the school premises using mouth mirror and CPI probe with the subject seated on a portable dental chair.

Subjects’ caries risk was assessed using computer based Cariogram based on the seven factors indicated in programme. The saliva and bacterial testing were excluded. Data for “caries experience” and “plaque content” were collected based on clinical observations using the aforementioned indices. Information on caries related systemic disease and exposure to fluoride were obtained from a parent-proxy questionnaire. For variable “clinical judgement”, decision was based on the overall scores of selected factors in cariogram and sociodemographic factors. A three-day diet diary was used to estimate the frequency of meals/snack intake per day and content of diet. Upon submission, the diet diaries were checked and a short interview with the subjects was conducted to ensure the accuracy of the information provided in the diet diary. When all information have been collected and inserted in the Cariogram, the computer programme categorised each child into a low, moderate, or high caries risk group.

Chi square test was used to evaluate the association of levels of caries risk with sociodemographic background and mean DMFT scores. Cross-tabulation was performed to estimate the proportions of children and the distribution of variables score occurrence according to the caries risk assessed. Linear and multiple regressions were performed to evaluate the predictability of caries risk. The level of significance was set at 0.05.

## RESULTS

A total of 300 children at the seven randomly selected schools in Bhakkar City fit into the inclusion criteria. However, only 226 agreed to participate, giving a response rate of 75%. The proportion of boys (54.4%) was slightly higher than girls (45.6%). Most (82.3%) lived in urban location and were from public funded schools (65.9%) (Table-I). Almost 40% of participants were categorised as having low risk for caries and number of those in the medium and high risk were almost equal. When the level of caries risk were assessed against the children’s sociodemographic background, it was found that those who went to private schools had higher number of children in the low caries risk group than those who went to public funded schools (p<0.001) (Table-I).

**Table-I:** Sociodemographic characteristics of respondents and their levels of caries risk (N= 226).

*Sociodemographic background*	*All n (%)*	*Level of caries risk according to Cariogram, n(%)*			*p-value*

		*Low*	*Moderate*	*High*	
All subjects		90 (39.8)	69 (30.5)	67 (29.7)	
***Gender:***					
Male	123 (54.4)	49 (39.8)	38(30.9)	36(29.3)	0.98
Female	103(45.6)	41(39.8)	31(30.1)	31(30.1)	
***Location of residence***					
Urban	186(82.3)	77(41.4)	53(28.5)	56(30.1)	0.34
Rural	40(17.7)	13(32.5)	16(40)	11(27.5)	
***School type:***					
Public funded	149(65.9)	31(20.8)	63(42.3)	55(36.9)	.000
Private	77(34.1)	59(76.6)	6(7.8)	12(15.6)	
***Age***					
11 year	95(42%)	35(36.8)	33(34.7)	27(28.4)	0.49
12 year	131(58%)	55(42)	36(27.5)	40(30.5)	

The overall mean DMFT score was 1.49(±0.63). The mean DMFT score of those in the low caries risk group was 0. There were not much differences in the mean DMFT score of those in the moderate or high caries risk group (1.33 vs 1.51) (Table-II). Children were more likely to be placed in the high risk group if they had high DMFT score and had no exposure to fluoride such as toothpaste or additional fluoride measures such as tablets, rinses or varnishes (Table-III). Children with poor snacking frequency were more likely to be grouped in the high-risk category as compared to those with poor diet content.

**Table-II:** Mean DMFT scores of different caries risk levels.

*Level of caries risk*	*Mean DMFT±SD*	*P value*
All	1.49±0.63	0.000
Low	0	
Moderate	1.33±0.51	
High	1.51±0.65	

**Table-III:** Distribution of Cariogram variables to the levels of caries risk.

*Risk categories*	*Low risk (n= 90)*				*Moderate risk (n= 69)*				*High Risk (n= 67)*			

Cariogram scores * [n(%)]											

	*0*	*1*	*2*	*3*	*0*	*1*	*2*	*3*	*0*	*1*	*2*	*3*
Caries experience	90 (100)	0	0	0	61 (88.4)	8 (11.6)	0	0	19 (28.4)	43 (64.2)	4 (6.0)	1 (1.5)
Related disease	90 (100)	0	0	0	69 (100)	0	0	0	67 (100)	0	0	0
Diet content	0	49 (54.4)	40 (44.4)	1 (1.1)	0	14 (20.3)	53 (76.8)	2 (2.9)	0	31 (46.3)	36 (53.7)	0
Sugar frequency	50 (55.6)	35 (38.9)	5 (5.6)	0	15 (21.7)	40 (58.0)	13 (18.8)	1 (1.4)	26 (38.8)	33 (49.3)	7 (10.4)	1 (1.5)
Plaque level	0	65 (72.2)	25 (27.8)	0	0	31 (44.4)	37 (53.6)	1 (1.4)	0	25 (37.3)	41 (61.2)	1 (1.5)
Fluoride programme	0	2 (2.2)	88 (97.8)	0	0	1 (1.4)	61 (88.4)	7 (10.1)	0	0	52 (77.6)	15 (22.4)
Clinical judgment	1 (1.1)	88 (97.8)	1 (1.1)	0	1 (1.4)	64 (92.8)	4 (5.8)	0	2 (3.0)	52 (77.6)	13 (19.4)	0

* score 0 denotes the lowest risk and score 3 denotes the highest risk for that particular Cariogram variable.

To establish the variables that influence caries risk most, a univariate model was applied to all variables (Table-IV). Only the variable ‘place of residence’ does not have a significant effect on caries risk profile, and hence this variable was excluded in the next analysis. All the other significant variables were included in the forward stepwise multivariate analysis and it was shown that the most relevant variable in caries risk prediction was previous experience of dental caries, which explains 40% of the caries risk observed (Table-V).

**Table-IV:** Variables included in linear regression model.

*Variables*	*p*
Caries experience	0.000
Diet content	0.008
Frequency of diet	0.002
Plaque scores	0.000
Fluoride sources	0.000
Clinical judgement	0.002
Type of school	0.000
Place of residence	0.144

**Table-V:** Variables in forward step wise multiple regression model.

*Step*	*Variable*	*F*	*β*	*p*	*R^2^*
1	Caries experience	148.5	0.568	0.000	0.40
2	Fluoride program	136.1	0.342	0.000	0.55
3	School	131.1	-0.323	0.003	0.63
4	Clinical Judgement	113.4	0.180	0.000	0.67
5	Plaque scores	99.8	0.167	0.001	0.69
6	Diet Frequency	92.4	0.115	0.009	0.71
7	Diet content	82.3	0.102	0.000	0.72

## DISCUSSION

Caries risk assessment involves the evaluation of caries diseases indicators, caries risk and protective factors to predict future dental caries and to determine the factors that contribute most to the caries incidence in the individual.11 Many validated CRA tools have been developed to assist oral health professionals in establishing the caries risk profiles of their patients. It is recommended that the selected CRA tool to be used is simple and cheap with finite equipment, and the technique employed is acceptable and comfortable for patients.6 In our study, the reduced Cariogram tool, where saliva and microbial tests were excluded, was used to evaluate the caries risk levels of our samples. By not including the aforementioned tests, the Cariogram ability to predict caries risk may be weakened.5 However, saliva and microbial analyses have been shown to have low predictive values in relation to dental caries and not practical for routine used in clinical practice. 12 In addition, in the presence of fluoride, caries causing microorganism may be tolerated in oral cavity without damaging the dentition.11 Hence, based on these factors, the reduced Cariogram ability to predict caries risk in our samples may not be fully jeopardized.

The main findings of our study shows that most 11-12 year old Pakistani school children were in the low caries risk group with almost absence of caries (DMFT equal 0). Even those in the high caries risk group had very low severity of caries with a mean score of 1.51. A possible explanation for this is that natural water fluoride is present in Bhakkar district and the fluoride concentration that ranges from 0.05 to 2.62 ppm may have an effect on the low incidence of caries in these children.13 As presence of water fluoridation is not one of the factor considered under the ‘fluoride sources’, this component was considered under the ‘clinical judgement’ variable by taking into account the children’s place of residence and its fluoride level in the water. Each factor in the Cariogram is weighted for its cumulative input14, but the programme places a high weightage on the ‘fluoride sources’ factor.11 As observed in our findings, children who did not use fluoride tooth paste or were not exposed to any other fluoride sources such as tablets, rinses or supplements, were categorized as having moderate or high risk for caries.

Of the four aforementioned factors that were relevant in determining the level of caries risk in our sample population, past caries experience was the most significant variable in the caries risk observed. This finding is similar in previous studies that used Cariogram programme on 10-11 year old school children in Sweden14 and Brazilian 7-9 year old school children.15 Indeed, epidemiological studies have shown a positive strong correlation between past caries experience and future caries development.12 The predictive power of this indicator is approximately 60%16 and past caries experience has been stated as the strongest single predictor of future carious lesions.17 The progression or regression of current caries on existing restorations depends on the exposure of the risk factors such as sugar diet and bacteria in saliva or plaque and the presence of protective factors such as fluoride and dental sealants. Hence, appropriate intervention therapy should be provided to high caries risk children and this include appropriate fluoride therapy, fissure sealant for their non-carious occlusal fissures, minimal intervention therapy for the cavitated lesions, and diet and plaque control counselling. Dental recalls for these children should be conducted every three months and radiographs taken every six months.18

## CONCLUSION

Most 11-12 year old Pakistani school children had low risk to caries with very low caries prevalence rate. The factor that largely predicts incidence of future caries in this study population was past caries experience. Appropriate caries management should be undertaken based on the children’s level of caries risk.
